# Complementarity of Rotating Video and Underwater Visual Census for Assessing Species Richness, Frequency and Density of Reef Fish on Coral Reef Slopes

**DOI:** 10.1371/journal.pone.0084344

**Published:** 2014-01-02

**Authors:** Delphine Mallet, Laurent Wantiez, Soazig Lemouellic, Laurent Vigliola, Dominique Pelletier

**Affiliations:** 1 IFREMER, Unité de Recherche Lagons, Ecosystèmes et Aquaculture Durable en Nouvelle Calédonie (LEAD-NC), Nouméa, New Caledonia; 2 EA 4243 LIVE, Université de la Nouvelle-Calédonie, Nouméa, New Caledonia; 3 Institut de recherche pour le développement (IRD), Laboratoire Excellence LABEX Corail, UMR 227 CoRéUs, Nouméa, New Caledonia; Leibniz Center for Tropical Marine Ecology, Germany

## Abstract

Estimating diversity and abundance of fish species is fundamental for understanding community structure and dynamics of coral reefs. When designing a sampling protocol, one crucial step is the choice of the most suitable sampling technique which is a compromise between the questions addressed, the available means and the precision required. The objective of this study is to compare the ability to sample reef fish communities at the same locations using two techniques based on the same stationary point count method: one using Underwater Visual Census (UVC) and the other rotating video (STAVIRO). UVC and STAVIRO observations were carried out on the exact same 26 points on the reef slope of an intermediate reef and the associated inner barrier reefs. STAVIRO systems were always deployed 30 min to 1 hour after UVC and set exactly at the same place. Our study shows that; (i) fish community observations by UVC and STAVIRO differed significantly; (ii) species richness and density of large species were not significantly different between techniques; (iii) species richness and density of small species were higher for UVC; (iv) density of fished species was higher for STAVIRO and (v) only UVC detected significant differences in fish assemblage structure across reef type at the spatial scale studied. We recommend that the two techniques should be used in a complementary way to survey a large area within a short period of time. UVC may census reef fish within complex habitats or in very shallow areas such as reef flat whereas STAVIRO would enable carrying out a large number of stations focused on large and diver-averse species, particularly in the areas not covered by UVC due to time and depth constraints. This methodology would considerably increase the spatial coverage and replication level of fish monitoring surveys.

## Introduction

Coral reefs and their adjacent ecosystems (mangroves, seagrass and algae beds, unvegetated soft bottoms, etc.) have high diversity, comparable to tropical rainforests [1]. Ecosystem services delivered by coral reefs are extremely important (fisheries, aquaculture, medicines, building material, tourism, etc.). Coral reefs are also an important natural protection for the coast and, in some parts of the world, human populations are closely related to coral reefs for their cultures and food supply. Nevertheless, despite their overt usefulness it is estimated that coral reefs have lost 20% of their area world wide due to human activity, especially in highly urbanized coastal areas. Globally 75% of reefs are currently threatened and 60% are immediately under direct threat [2].

Estimating diversity and abundance of fish is fundamental for understanding community structure and dynamics of coral reefs. When designing a sampling protocol, one crucial step is the choice of the most suitable sampling technique which is a compromise between the questions addressed, the available means and the precision required [3], [4], [5]. Therefore it is essential to compare the capacity of each technique to estimate biodiversity and abundance in order to disentangle the differences due to the technique used from real spatial or temporal patterns.

In the marine environment, capture techniques are generally used to estimate the abundance of species and can be performed with explosives/ichtyocides [3], [6], trapping [7], [8], trawling/netting [9], [10] and hook and line [11]. Direct observation techniques include Underwater Visual Census (UVC), video, acoustics [12], [13] and photographic techniques [14], [15]. Direct observation techniques have been used to estimate diversity and abundance of marine organisms. In coral reefs, UVCs are by far the most commonly used technique, and include strip transects [16], line transects [17], rapid visual census [18] or stationary point counts [19]. However, video techniques are increasingly used based on either mono [20] or stereo cameras [21] and are either unbaited [22] or baited [23] with systems either operated by divers [24], towed [25] or remote [26].

With respect to coral reefs, many studies compared UVC and video techniques (whatever the techniques used) but few examine the remote underwater video technique (RUV which is remote, unbaited, not-towed and not operated by a diver). Francour et al. [27] and Burge et al. [28] compared RUV with the stationary point count UVC technique in the same area. They compared the species richness and abundance observed between the two techniques, but observations were not performed at the same locations or at concurrent times. They both showed that the overall species richness and abundance recorded were higher for UVC than for RUV. To our knowledge there is no study comparing observations obtained by RUV and UVC using the same sampling strategy that can be considered as paired (less than an hour difference between observations, same day and location).

The objective of the present study is to compare the ability of two techniques, currently used in New Caledonia, to sample reef fish communities: the UVC stationary point counts technique [19], [29] and the rotating video technique (STAVIRO for French “STAtion Video ROtative) [30]. For both techniques, fish are counted over 360° at fixed points. This study was conducted during a regular annual survey of coral reefs within a marine protected area using the UVC stationary point counts technique. Comparisons were performed at the same locations, where both techniques were usable, on two types of reef (intermediate and inner barrier) in order to investigate the following hypotheses: (1) there will be significant differences between each technique in the resulting fish assemblage data; (2) a greater number of small species is sampled with UVC; (3) target species are better observed with STAVIRO.

## Materials and Methods

### Ethics Statement

No specific permits were required for the described field studies. During the field study, only the video systems and divers were immersed in water; no animals (including endangered or protected species) were collected or manipulated. Field work did not require any permission in the study area; it was accomplished with the approval of the Direction de l’Environnement of the South Province in charge of managing the study area. Our field work activites fully complied with New Caledonian environmental regulations (Code of the Environment, http://www.province-sud.nc/images/stories/pdf/environnement/Code.pdf).

### Sampling Protocol

**Figure 1:**
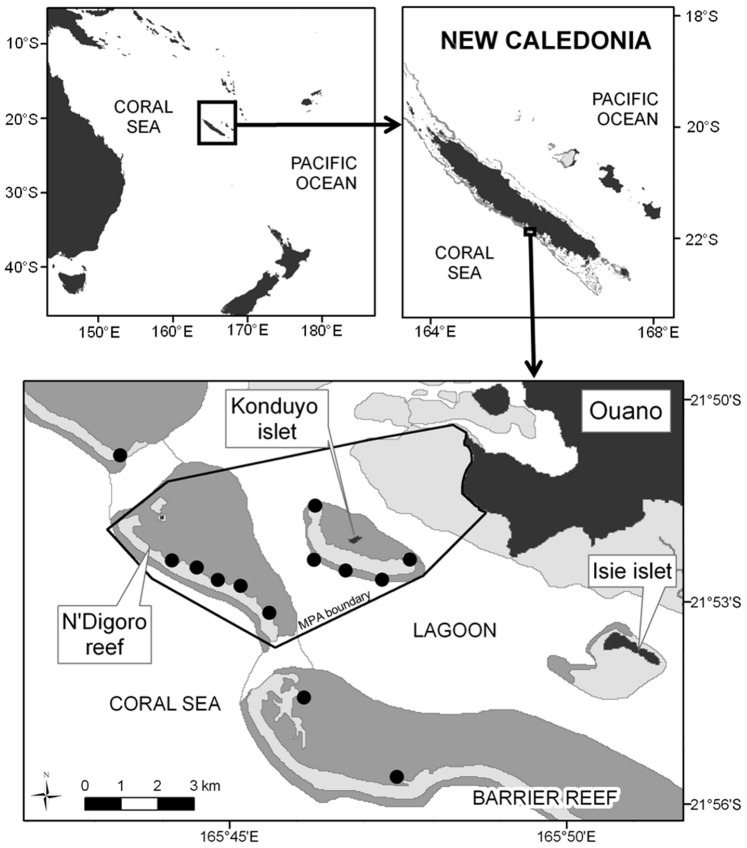
Study area. Each black dot contains 2 censused points (one on the top of the reef slope and one on the bottom of the reef slope).

The study was conducted, from 5^th^ to 16^th^ October 2009, in the Southwest Lagoon of New Caledonia, South Pacific. The study area encompassed the MPA of Ouano (21°50′S, 165°45′E) and the nearby unprotected areas. This MPA was created in 2004 and rules have been enforced since 2007. Three lagoon-reef components (fringing reef, intermediate reef and barrier reef), seagrass beds and mangroves are present in this study area. UVC and STAVIRO observations were carried out on the exact same 26 points on the reef slope of the intermediate reef and the inner barrier reefs (Fig. 1). In order to avoid the known influence of divers on fish behavior [31], [32], [33], STAVIRO systems were always deployed 30 min to 1 hour after UVC. Once their counts were completed, divers left a weighted buoy to mark sampling sites, so that STAVIROs were set at the exact same place.

### Data Collection

The STAVIRO technique used for this study is autonomous, remote, unbaited, and rotates by 60° each 30 sec. Videos are recorded without any external disturbance for around 10 min. at each site, with at least three complete rotations [30].

The UVC technique used is a modification of the point count technique [19]. All observed species are recorded by 2 stationary divers back to back, over a 10 minute period of time with in a virtual cylinder extending from surface to bottom. One diver sampled mobile species and the other sampled small species. A measuring tape was set on the sea bottom before performing the counts to validate distance estimates only for UVC.

Due to the protocol constraints of each technique, small species were identified in a virtual cylinder of 3 m radius for UVC [29], [34] and 5 m radius for STAVIRO [30], and large species were identified within the visibility range limited to a maximum of 10 m for both techniques. A species was considered as “small” when the maximum species size was less than 30 cm (see Table S1 for the list of small and large species).

### Species Identification

For both techniques, all individuals were identified at the highest possible taxanomic level. Species of the same genus, which differed by a characteristic that was difficult to distinguish on video images particularly beyond a few meters from the camera (blue eyes, small black dot, etc.), were aggregated only for the STAVIRO analyses as (i) *Stegastes gp* for *Stegastes fasciolatus*/*Stegastes nigricans*/*Stegastes punctatus*; (ii) *Ctenochaetus gp* for *Ctenochaetus striatus*/*Ctenochaetus binotatus*/*Ctenochaetus cyanocheilus*; (iii) *Acanthurus gp* for *Acanthurus blochii*/*Acanthurus dussumierii* and (iv) *Kyphosus gp* for *Kyphosus vaigiensis*/*Kyphosus cinarescens*/*Kyphosus sydneyanus*. These species were not aggregated for the UVC technique as divers were fully confident about their identifications.

### Images Analysis

A single observer analyzed all videos using the procedure explained in Pelletier et al. [30]. Individuals were counted per sector and then summed over the six sectors of a rotation (360°). Thus, for each station and species, three counts were obtained, each corresponding to one of the three rotations analyzed. Then, abundance per species at a given station was calculated as the maximum count taken over the three rotations. To minimize potential double counting from one sector to another, particular attention was given to the direction of fish movement with respect to camera rotation. For the STAVIRO technique, distances were estimated with previously-taken footage using a database of screenshots of plastic fish silhouettes of several sizes (0.2 m, 0.4 m, 0.6 m, 0.8 m and 1 m) and colors (bright and dark ones), taken at several distances from the same camera (2 m, 5 m, 7 m and 10 m). This method has been proven to be helpful to estimate distances on footage, even if the screenshots used were not taken in the study area [35].

### Statistical Analysis

We first compared the species assemblages observed by each technique using the Sørensen Index (S) where S = 2a/(2a+b+c) with a = number of species observed by both methods; b = number of species not observed with STAVIRO; and c = number of species not observed with UVC [36]. Pearson correlation tests were then used to compare species richness (SR) per station (number of species observed), fish density (number of individual per square meter), number of genus and families observed by each technique per station. The factor “species size” referred to maximum species size and was qualified as “large” or “small” in the following parts (see Table S1 for details). The influence of species size on observed species richness per station and density was analysed using 2-way repeated measures ANOVA (same site sampled by the 2 techniques) with factors species size (small or large) and technique (UVC and STAVIRO) as fixed factors. The homoscedasticity of variances was obtained on log transformed data (Levene’s test for homogeneity of variance, p>0.05; [37]). When interactions between techniques and species size were significant, differences between techniques according to species-size were further tested using pairwise comparison tests performed at α = 0.05.

Paired Student t-tests were then used to compare observations between techniques (SR per station and density depending on size of species observed) for commonly seen families (i.e. encountered in more than 50% of stations for both techniques).

We assessed the ability of each technique to observe target species by analysing the effect of a species being a fishery target (target or non-target, see Table S2 for the list of target species in New Caledonia) on observed species richness and density. This was achieved by 2-way repeated measures ANOVA with factors fishery target (target or non-target) and technique (UVC and STAVIRO) as fixed factors. The homoscedasticity of variances was obtained on square root transformed data (sqrt(x+1)) for species richness and on log transformed data (log(x+0.1) for the density (Levene’s test for homogeneity of variance, p>0.05; [37]). When interactions between technique and fishery target were significant, differences between techniques according to fishery target were further tested from pairwise comparison tests between group levels performed at α = 0.05.

In the study area, assemblage structure may differ according to reef habitat and environmental factors. We investigated whether the techniques detected similar assemblage structures using a Factorial Correspondence Analysis (FCA; [38]) performed on densities per species and station. A Hierarchical Ascending Classification (HAC; [37]) was performed on the first seven axes of the FCA. These extracted more than 50% of total inertia (52% for UVC and 54.5% for STAVIRO). The HAC used the Euclidean distance and the aggregation method of Ward [38]. For each of the assemblages defined, a species was deemed characteristic of the assemblage when its relative contribution to the first seven axes of the correspondence analysis was higher than 30%.

## Results

### Fish Identification and Detection

With the STAVIRO technique, 1941 individuals corresponding to 118 species, 63 genera and 30 families, were counted (Table 1). 121 (i.e. 6.2%) individuals were identified only at the genus level and 129 (i.e. 6.6%) only at the family level. For the vast majority of these (119 Scaridae, 48 Pomacentridae, 37 Caesionidae, 13 Acanthuridae, 10 Chaetodontidae, 8 Lethrinidae, 5 Labridae, 3 Serranidae, 3 Sphyraenidae, 2 Mullidae, 1 Blenniidae and 1 Haemulidae), the fish were either too far away, too small or swam through the field of the camera too quickly to be identified at the species level.

**Table 1:** Overall abundance, species and genus number per family observed by each technique.

	STAVIRO				UVC			
Numbers observed	Freq	ind	sp	ge	Freq	ind	sp	ge
**Acanthuridae**	**96.2**	**175**	**10**	**4**	**96.2**	**189**	**13**	**4**
Apogonidae	0	0	0	0	11.5	5	4	2
Aulostomidae	7.7	2	1	1	0	0	0	0
**Balistidae**	**57.7**	**29**	**3**	**3**	**65.4**	**32**	**3**	**3**
Blenniidae	7.7	2	1	1	30.8	20	5	4
Caesionidae	23.1	98	2	2	19.2	141	2	1
Carangidae	3.8	1	1	1	3.8	1	1	1
Carcharhinidae	7.7	2	1	1	0	0	0	0
**Chaetodontidae**	**88.5**	**84**	**15**	**3**	**100**	**115**	**19**	**3**
Cirrhitidae	0	0	0	0	15.4	5	2	2
Diodontidae	7.7	2	2	1	0	0	0	0
Fistulariidae	3.8	1	1	1	0	0	0	0
Gobiidae	3.8	1	1	1	15.4	5	2	2
Haemulidae	3.8	1	0	0	3.8	3	2	2
Holocentridae	0	0	0	0	3.8	18	0	1
Kyphosidae	3.8	14	1	1	0	0	0	0
**Labridae**	**100**	**131**	**20**	**11**	**100**	**245**	**30**	**15**
Lethrinidae	46.2	62	5	3	30.8	65	3	4
Lutjanidae	15.4	58	3	2	23.1	99	4	1
Monacanthidae	7.7	3	2	2	11.5	5	1	1
**Mullidae**	**65.4**	**78**	**5**	**1**	**73.1**	**53**	**6**	**1**
**Nemipteridae**	**76.9**	**154**	**2**	**1**	**80.8**	**130**	**3**	**1**
Ostraciidae	3.8	1	1	1	3.8	1	1	1
Pinguipedidae	15.4	4	2	1	38.5	13	4	1
**Pomacanthidae**	**34.6**	**15**	**2**	**1**	**57.7**	**29**	**4**	**2**
**Pomacentridae**	**88.5**	**656**	**15**	**9**	**96.2**	**604**	**29**	**10**
Priacanthidae	7.7	12	1	1	11.5	5	1	1
**Scaridae**	**88.5**	**286**	**13**	**3**	**76.9**	**132**	**12**	**3**
**Serranidae**	**19.2**	**28**	**3**	**3**	**53.8**	**39**	**5**	**3**
Siganidae	23.1	35	3	1	38.5	61	5	1
Sphyraenidae	3.8	3	0	1	3.8	100	1	1
Stegostomatidae	3.8	1	1	1	0	0	0	0
Synodontidae	0	0	0	0	3.8	2	1	1
Tetraodontidae	7.7	2	1	1	19.2	7	1	1
Total		1941	118	63		2124	164	73

Freq: Frequency (in %); ind: number of individuals; sp: number of species and ge: number of genera. Families with frequency higher than 50% are shown in bold.

With the UVC technique, 2124 individuals, corresponding to 164 species, 73 genera and 28 families were counted (Table 1). 167 (i.e. 7.9%) individuals were identified only at genus level and none were identified only at family level. Among the 167 individuals only identified at genus level, 59 were Scaridae (mainly juveniles), 40 Acanthuridae, 28 Lethrinidae, 20 Siganidae, 18 Holocentridae, 1 Labridae and 1 Serranidae. Individuals were either too far away or hidden in holes in the reef substrate to be identified at the species level.

In all, 194 distinct species were observed with either technique, 88 (i.e. 45.8%) being observed with both techniques, 76 (i.e. 39.6%) only with UVC and 28 (i.e. 14.6%) only with STAVIRO. Among the 76 species only observed with the UVC technique, 14 were cryptic (Apogonidae, Blenniidae, Cirrhitidae and some Chaetodontidae and Pomacentridae), 20 species were too small to be identified with the video technique (15 species of Pomacentridae and 5 species of Labridae), while others were only seen with the UVC technique due to sampling variability as observations were not performed simultaneously, such as some Labridae, Lethrinidae and Siganidae. Some species could be identified with the UVC technique but not with STAVIRO although they were recorded with the video technique. This was probably the case, for instance, for many Scaridae, Pomacentridae and Caesionidae identified with the STAVIRO technique at species (“sp.”) and genus (“ge.”) levels only. For instance, 16 species of Pomacentridae were identified with the UVC technique and not with the STAVIRO technique, while 48 individuals of Pomacentridae were recorded with STAVIRO but not identified at species level during the image analyses.

Among the 28 species only recorded with STAVIRO, 6 were shy and may have avoided divers, such as some *Lethrinus* spp. The other species only observed with the STAVIRO technique were mostly due to sampling variability such as some *Diodon* spp., *Scarus* spp., *Siganus* spp. and Labridae.

The Sørensen index computed on paired stations ranged from 0.1 to 0.53, with mean 0.37 and Standard Error (SE) 0.1, indicating that species sampled with each technique were different. At each station, roughly one third of the species was observed with both techniques, one third was only observed with UVC and one third was only observed with STAVIRO.

### Species Numbers and Overall Density

Correlation between observations with each technique at the station level was low but significant for species richness (R = 0.54, p-value <0.01), the number of genus and families (R = 0.64 and 0.73 respectively, p-values <0.01) and fish densities (R = 0.54, p-value <0.01).

**Figure 2:**
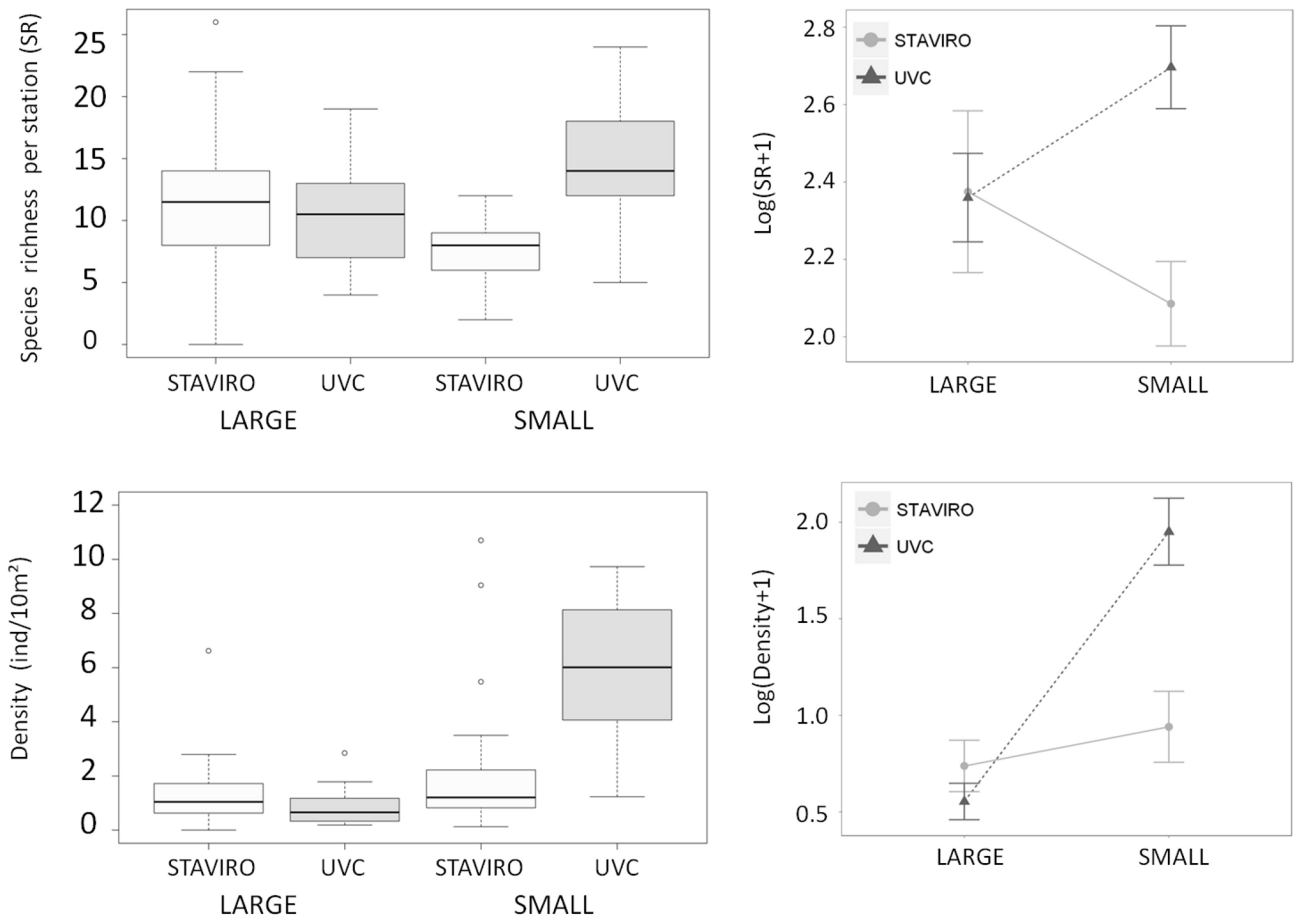
Species richness and density (number of individuals per 10 m^2^) observed per station according to technique and species size. Three outlying values were not reported for better readability of the density plot: 14.85, 17.33 and 23.52 ind/10 m^2^ for small UVC. On the right of each boxplot, interaction plots on log-transformed averages are shown.

The two-way ANOVAs with factors technique and species size displayed a significant interaction between factors for species richness (p<0.01) (Fig. 2 top), with more small species recorded with UVC (mean ± SE = 14.58±0.91) than with STAVIRO (mean ± SE = 7.46±0.47) (pairwise comparisons test, p<0.01). On the other hand, the number of large species was not significantly different between techniques (pairwise comparisons test, p>0.05). However, on average a little more large species were observed with STAVIRO (mean ± SE = 11.50±1.14) than with UVC (mean ± SE = 10.23±0.73).

Regarding density, interaction between technique and species size was significant (2-way ANOVA, p<0.01) (Fig. 2 bottom). With the UVC technique more individuals were recorded from small species (mean ± SE = 7.14±0.97 ind/10 m^2^) than with the STAVIRO technique (mean ± SE = 2.13±0.50 ind/10 m^2^) (pairwise comparisons test, p<0.01). In contrast, the number of individuals from large species did not significantly differ between techniques (pairwise comparisons test, p-value >0.05), although on average more individuals from large species were identified with STAVIRO (mean ± SE = 1.31±0.25 ind/10 m^2^) than with UVC (mean ± SE = 0.82±0.12 ind/10 m^2^).

### Most Frequent Families

**Figure 3:**
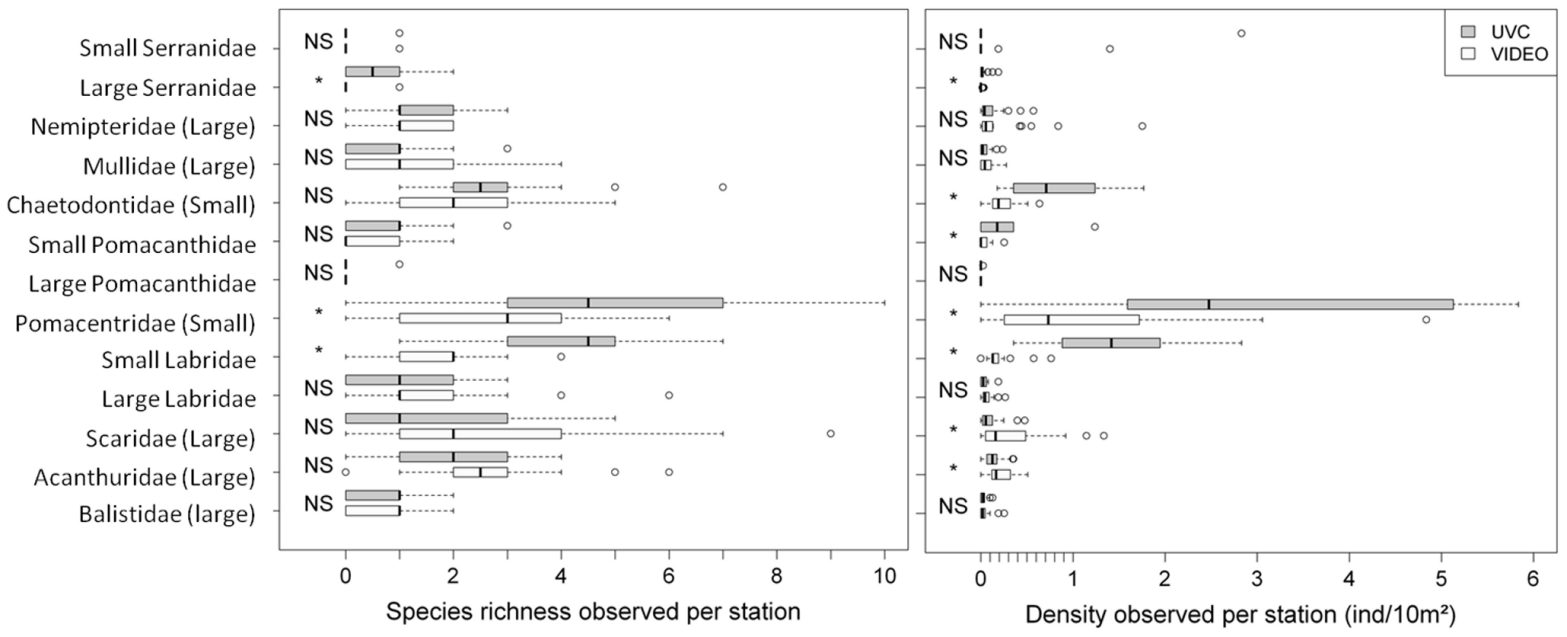
Species richness and density observed per station for the 10 main families observed, distinguishing the size class of the species observed as small or large (see previous paragraph). A size class in brackets indicates that all species observed in the family are characterized by the same size group. The result of the Student t-test on the difference between techniques is reported on the left side of the plot. NS: p>0.05; *: p<0.05.

Species richness per station did not differ significantly between techniques for small Serranidae, Nemipteridae, Mullidae, Chaetodontidae, large Pomacanthidae, large Labridae, Scaridae, Acanthuridae and Balistidae (paired t.test, p>0.05) (Fig. 3 left). For large Serranidae, Pomacentridae and small Labridae, significantly more species per station were observed with the UVC technique (paired t.test, p<0.05) (Table 2). Densities were not significantly different between techniques for small Serranidae, Nemipteridae, Mullidae, large Pomacanthidae, large Labridae and Balistidae (paired t.test, p>0.05) (Fig. 3 right). Chaetodontidae, small Pomacanthidae, Pomacentridae and small Labridae densities were significantly higher for UVC (Table 3). In contrast, densities of Scaridae and Acanthuridae (which comprise only large species) were significantly greater for STAVIRO (paired t.test, p<0.05) (Table 3).

**Table 2:** Mean number of species per station (±SE) recorded for main families observed with UVC and STAVIRO.

	STAVIRO	UVC
Serranidae (Small)	0.04±0.06	0.04±0.06
Serranidae (Large)	**0.12±0.10**	**0.58±0.20**
Nemipteridae (Large)	1.12±0.24	1.31±0.27
Mullidae (large)	1.31±0.39	1.04±0.28
Chaetodontidae(Small)	1.96±0.43	2.92±0.53
Pomacanthidae (Small)	0.42±0.20	0.73±0.25
Pomacanthidae (Large)	0.00±0.00	0.04±0.06
Pomacentridae (Small)	**2.81±0.57**	**4.89±0.88**
Labridae (Small)	**1.85±0.28**	**4.19±0.53**
Labridae (Large)	1.58±0.43	1.19±0.53
Scaridae (Large)	2.42±0.72	1.77±0.52
Acanthuridae (Large)	2.65±0.45	1.96±0.34
Balistidae (Large)	0.69±0.22	0.73±0.19

Significant differences between techniques according to Student t-test are in bold (p<0.05).

**Table 3:** Mean density (±SE) (ind/m^2^) recorded for main families observed with UVC and STAVIRO.

	STAVIRO	UVC
Serranidae (Small)	0.06±0.09	0.11±0.18
Serranidae (Large)	**0.01±0.01**	**0.03±0.01**
Nemipteridae (Large)	0.19±0.12	0.10±0.05
Mullidae (large)	0.07±0.03	0.04±0.02
Chaetodontidae(Small)	**0.21±0.05**	**0.78±0.16**
Pomacanthidae (Small)	**0.04±0.02**	**0.19±0.08**
Pomacanthidae (Large)	0.00±0.00	0.01±0.01
Pomacentridae (Small)	**1.61±0.75**	**4.11±1.49**
Labridae (Small)	**0.19±0.05**	**1.41±0.21**
Labridae (Large)	0.06±0.02	0.04±0.01
Scaridae (Large)	**0.33±0.12**	**0.10±0.04**
Acanthuridae (Large)	**0.20±0.04**	**0.15±0.03**
Balistidae (Large)	0.04±0.02	0.03±0.01

Significant differences between techniques according to Student t-test are in bold (p<0.05).

### Observation of Target Species

**Figure 4:**
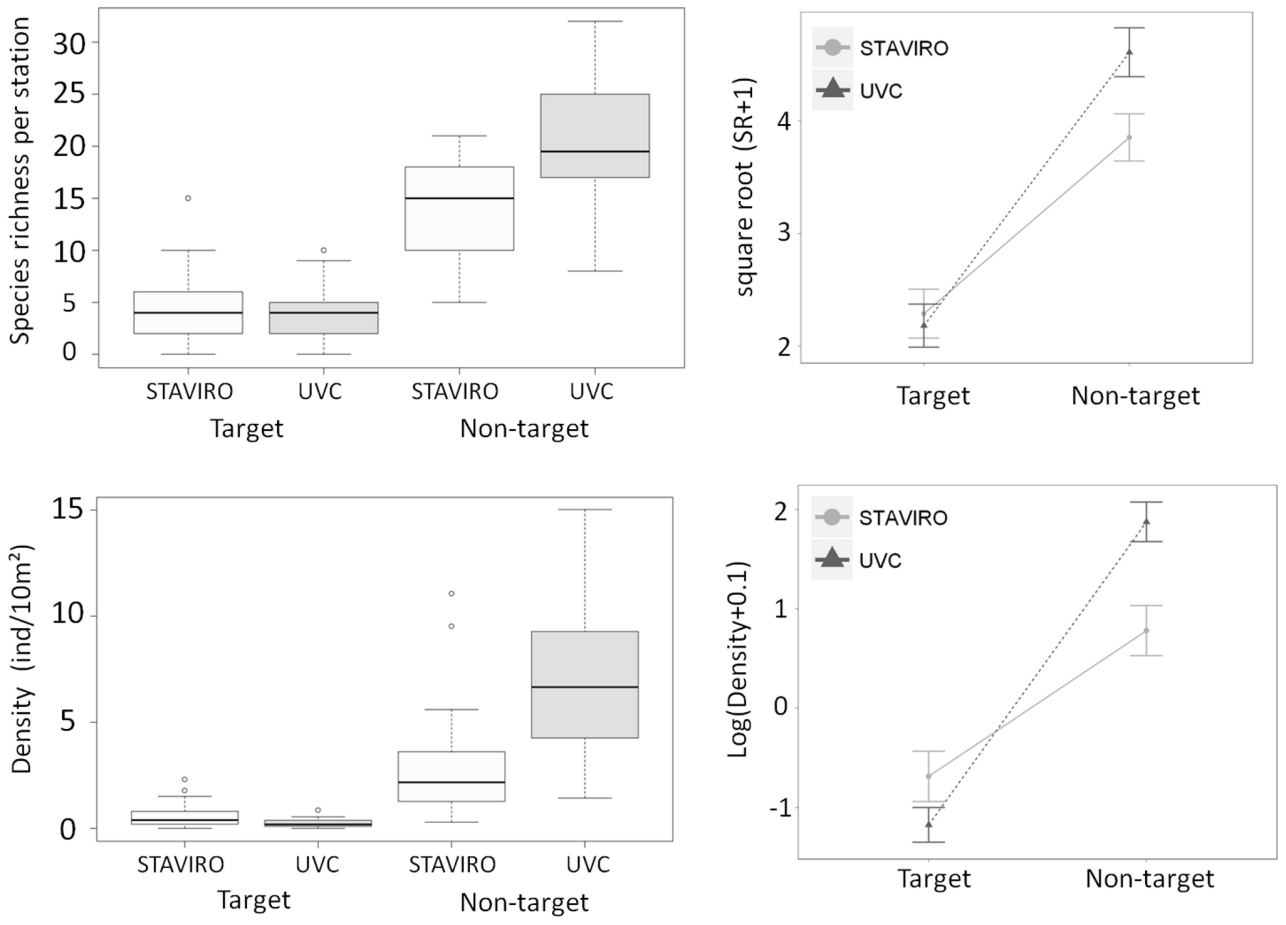
Species richness and density (number of individuals per 10 m^2^) observed per station according to technique and to fishery target. Two outlying values were not reported for better readability of the density plot: 17.48 and 23.84 ind/10 m^2^ for non-target UVC. On the right of each boxplot, interaction plots of log- and x^2^-transformed averages are shown.

The two-way ANOVAs with factors technique and fishery target displayed a significant interaction for species richness (p<0.01), indicating that more non-target species were recorded with the UVC technique (mean ± SE = 20.69±1.20) than with the STAVIRO technique (mean ± SE = 14.27±0.94) (pairwise comparisons test, p<0.01) (Fig. 4 top). The number of target species observed per station did not significantly differ between techniques (STAVIRO: mean ± SE = 4.69±0.66, and UVC mean ± SE = 4.12±0.52) (pairwise comparisons test, p>0.05).

Regarding densities, the two-way ANOVA led to a significant interaction between factors (p<0.01), indicating that more individuals from non-target species were recorded with the UVC technique (mean ± SE = 7.71±0.97 ind/10 m^2^) than with the STAVIRO technique (mean ± SE = 2.86±0.51 ind/10 m^2^) (pairwise comparisons test, p<0.01) (Fig. 4 bottom). In contrast, more individuals from target species were recorded with the STAVIRO technique (mean ± SE = 0.57±0.11 ind/10 m^2^) than with the UVC technique (mean ± SE = 0.26±0.04 ind/10 m^2^) (pairwise comparisonstest, p<0.05).

The fished species observed with each technique were all large species and indeed not the same. Only 17 species were observed with both UVC and STAVIRO: 3 species of Acanthuridae, 1 Labridae, 1 Mullidae, 1 Priacanthuridae, 9 Scaridae, 1 Serranidae and 1 Siganidae. Target species only observed with the STAVIRO technique (10 species) were composed of 1 species of Acanthuridae, 1 Carangidae, 1 Kyphosidae, 4 Lethrinidae and 3 Scaridae, and target species only observed with the UVC technique (15 species) were composed of 4 species of Acanthuridae, 1 Carangidae, 1 Haemulidae, 3 Scaridae, 2 Serranidae and 4 Siganidae.

### Do the Techniques see Similar Assemblages?

**Figure 5:**
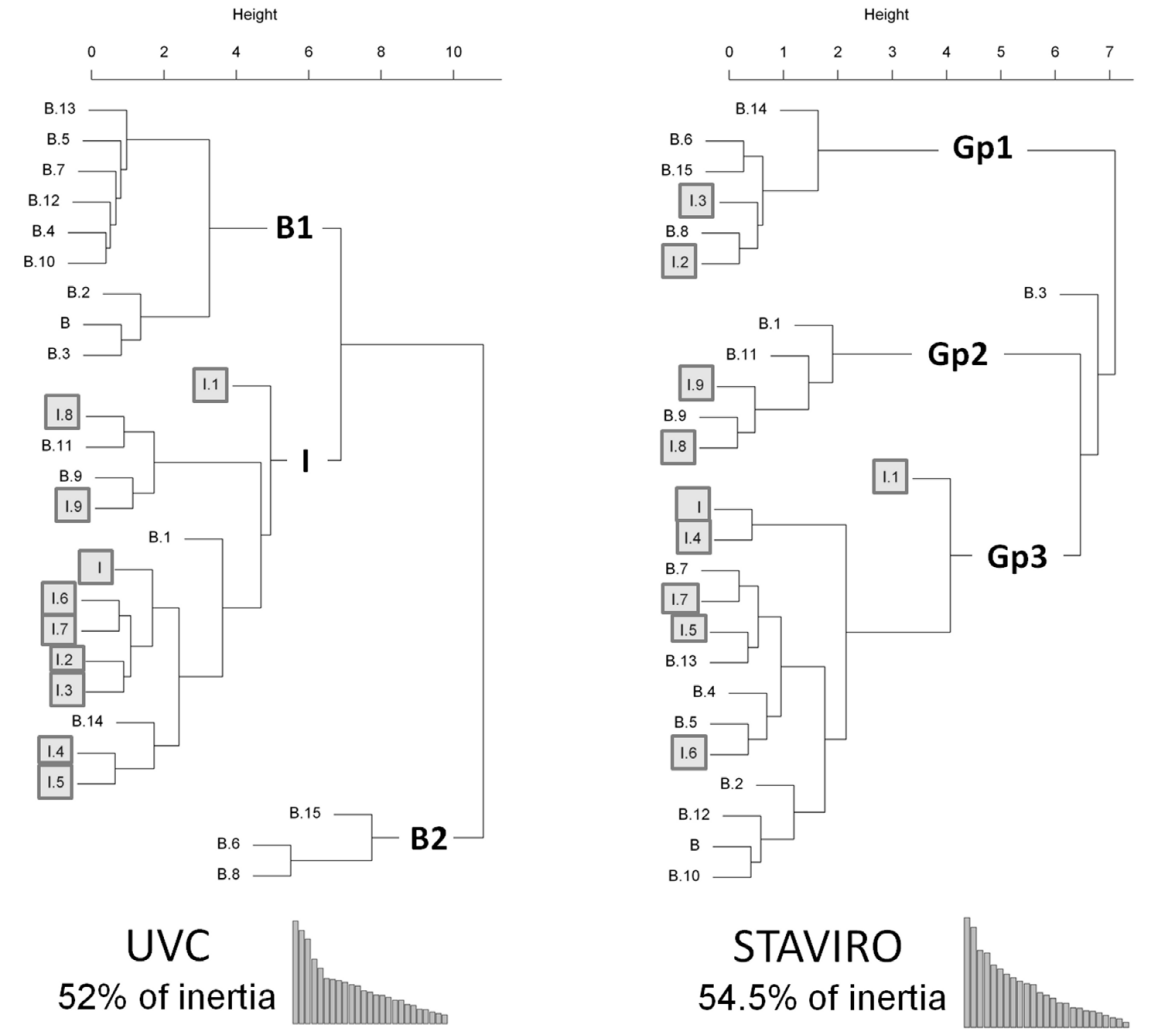
Dendrogram of the stations. B: Barrier reef and I: Intermediate reef. Clusters are represented on the graph in bold: B1 = 1st cluster on the barrier reef; I = cluster on the intermediate reef; B2 = 2nd cluster on the barrier reef; Gp1, 2 and 3 = clusters not explained by habitat characteristics. Intermediate reef stations are in grey and barrier reef stations are in white.

For the UVC technique, three assemblages could be identified from the FCA and HAC: one intermediate reef community and two barrier reef communities (Fig. 5 left). The intermediate reef community (group I) was characterized by fish species associated with lagoonal coral reefs. These species were all small, mostly sedentary (1 mobile and 3 sedentary species) and only observed with UVC (see Table S3 for the list of species characterizing each group). The first barrier reef community (group B1) was characterized by species usually found at the back of the inner barrier reef with rock, rubble and algae. These species were (1) as much sedentary as mobile (10 sedentary and 8 mobile species), (2) equally large and small (11 large and 8 small species), and (3) 9 species were observed with both techniques and 9 other species were only observed with UVC technique. The second barrier reef community (group B2) was characterized by fish species associated with branching *Acropora*. These species were (1) more sedentary than mobile (7 sedentary and 3 mobile species), (2) equally large and small species (5 large and 5 small species), and (3) 6 species were observed with both techniques and 4 other species were only observed with UVC technique. Four stations from the barrier reef were included in the group I which means that the assemblages observed on these stations were closer to the intermediate reef assemblage than the inner barrier reef ones.

With the STAVIRO technique no similar organization was highlighted at this spatial scale (Fig. 5 right). As for UVC, the analysis also shows the existence of three different groups with 54.5% of inertia (compared to 52% of inertia for UVC) and one single particular station (B.3: station from the inner barrier reef). Compared to UVC, these groups have no spatial organization that can be related to an ecological pattern in the area, both spatially and geomorphologically. Each group contains a mix of intermediate (from 33% to 43%) and barrier (from 57% to 67%) reefs.

## Discussion

Many studies have compared UVC and video techniques, and in particular strip transect UVC and baited underwater video system (BRUV) [28], [39]–[45], except Lowry et al. [46] who used the stationary point count technique for UVC. Other studies compared strip transects or point counts UVC with (i) remote underwater video system (RUV) (stereo or mono) [27], [28], [47]–[53], (ii) towed video system (TOWV) [43], [54], [55], and (iii) diver-operated video (DOV) [56]–[63].

Many previous studies (whatever the technique used) did not conduct sampling at the same time and at the same place. If the comparison is focused on isolating the influence of diver on observation, apart from DOV, it is not possible to simultaneous conduct UVC and video activities. Therefore, most comparisons were not paired in space (e.g. [44] and [53]), or in time (e.g. Colton and Swearer [45] had from 1 to 39 days between BRUV and UVC censuses), and relied on different observation durations (e.g. [41] and [62]), or different observed surface areas (e.g. [54]). The most common finding of these published comparisons between the different observational techniques is that no single technique provides representative information on all fish species [39], [55], [64]. In the present study, observations were both paired in time (one hour lag between the UVC and the STAVIRO), space and duration.

### Techniques and Species Identification

From the data available (26 paired stations on the reef slope), about 30% more species were identified with UVC than with STAVIRO (164 vs. 118). More than half of the 76 species solely identified with UVC were small and sedentary (46 species), while the rest was large and mobile. In contrast, 24 out of the 28 species only identified with STAVIRO were large and mobile. At the station scale, thespecies assemblages recorded with STAVIRO and UVC also differed, as shown by relatively low correlations and Sørensen Indices. Thus, the two techniques used together allowed to observe a combined species richness of 194 species (compared to 164 or 118 for each technique alone), and with only 45% of speciesrecorded with both techniques (88 out of 194 species).

Difference in species identification can be partially explained by the techniques used. Tillett et al. [65] highlighted that, irrespective of the technique used, species identification is not always possible, and this is especially true when fish morphologies are similar. This effect is amplified when using autonomous underwater video systems since a close examination of details that are essential to discriminate some species with confidence is constrained by video quality and zooming performance during image analysis. In contrast, divers have the possibility to take a very close look at individuals difficult to identify. This could explain why many more small species were identified with UVC than with STAVIRO. Moreover, Francour et al. [27] observed that underwater perception by a diver was better than the one recorded by a video camera. Having a better 3D-vision underwater could also explain the difference in species identification between the two techniques as well as the large number of individuals identified only at genus level from video (2D-vision) [66].

Differences between the two techniques may also be explained by protocol constraints. The present study was coupled with a regular UVC survey. STAVIRO stations had to be implemented after UVC at prescribed locations of the routine monitoring. The STAVIRO technique has therefore not been used to its optimum capacity (choice of station location and with the usual level of spatial replication). In a comparative study of UVC and DOV transects, Pelletier et al. [62] found no significant effect of carrying out DOV before or after UVC, but both techniques were diver-based. In the present comparison, only one technique requires a diver. The effect of conducting UVC before STAVIRO was not formally tested, but did not seem to affect the STAVIRO sampling as we did not observe any correlations between observations. Thus, 1 hr time lag seemed a reasonable compromise to avoid the confounding effect of divers. However, we suggest that future comparison studies should attempt to randomize the order of the methods to formally test this possible bias.

Many studies have examined the response of fishes to diver presence, but to our knowledge none investigated the effect of an unbaited video system upon fish behavior. The presence of a video system underwater could also influence fish behavior. However, fish are not commonly exposed to this type of intrusion compared to divers who are increasingly present in the coastal environment [67]. Therefore, even if the system can influence the behavior of some fishes, this influence should be less intrusive than divers and should be the same in all habitats. Independently of fish behavior, observer effects have been studied many times including diver swim speed and search intensity [68], observer experience, and training level [69], [70]. For STAVIRO, the only possible observer effect is during the images analysis. Compared to UVC, video techniques have the advantage of generating images visible by different experts, at different times and support the comparison between observers for the same videos.

### Differences in Observations According to Species Size and Fishery Target

With regard to species size, UVC technique appeared more effective to census small species, both in terms of species numbers and densities. Regarding large species, the 2 techniques did not record the same species (43 species in common, 30 species only seen with UVC and 24 species only seen with STAVIRO) but appeared similarly effective, in terms of number of species and individuals sampled, as our results show no significant difference between techniques for the species richness per station and density observed for large species. The abilities of each technique to census small or large species can be associated with the ability of each technique to see underwater. The better perception of divers underwater can explain their enhanced ability to observe, count and identify small species. Our results are consistent with Bozec et al. [71] who studied the detection distance of reef fish from a large number of UVC transects performed in New Caledonia and French Polynesia. They showed that fish body size is the primary factor in determining fish detection. Individuals smaller than 30 cm are not well observed at distances larger than 4 m and larger fish are better observed beyond 3 m due to diver avoidance (large fish tending to be diver-averse). In our study, all individuals from the small species class were smaller than 30 cm (see Material and Methods) and therefore more difficult to observe as distance increases. Small species were not censused over the same area by the 2 techniques (3 m radius for UVC and 5 m radius for STAVIRO). Therefore, if species were detected with both techniques within their respective observation radius, density estimates should be comparable and species richness should be higher for STAVIRO. But, small species being less well detected at large distances, video density and species richness estimates of small species were smaller. Indeed, the STAVIRO protocol yielded a list of 26 families comprising mostly large species (including all fished species), Chaetodontidae, emblematic fish species, turtles, and Dugondidae (see [30] for details). In the present analysis, to produce a complete comparison between the two techniques, images were analyzed taking into account all species, and therefore, including small species within a radius of 5 meters. Our results show that image resolution does not allow the observation of all small species at such distance. For a better observation of small species, e.g. in specific ecological studies, the video systems need to be positioned closer to the species (e.g. [72]), with a reduced radius of observation.

With regard to fishery target species, UVC was found to observe more species and individuals from non-target species than STAVIRO. This outcome is consistent with results on species size, as many small species are not targeted by fishery. In contrast, more individuals from target species were observed with STAVIRO than with UVC technique. This could be due to the fact that some species may avoid divers’ presence [40], [73]. Note however that the target species observed by each technique were not the same, with only one third of species observed by both techniques over all stations. The low density of target species observed (STAVIRO mean = 0.58 ind/10 m^2^ and UVC mean = 0.26 ind/10 m^2^) could be explained by past fishing pressures in the area even if the study was conducted in an MPA. STAVIRO also censused more target species inhabiting lagoon bottoms (Lethrinidae). Januchowski-Hartley et al. [33] showed that spear fishing pressure influences the behavior of targeted reef fish and that these behavioral changes varied according to family.

Our results are consistent with previous studies that showed overall greater species richness and abundance observed by UVC compared to video technique ([27] for RUV vs UVC; [60], [62] for DOV vs UVC; [42], [44]–[46] for BRUV vs UVC). Our study details which species are better detected on lagoon reef slopes and explain differences by species size and fishery target. In addition, since observations are truly paired, other sources of variability are minimized.

### Consequences at Family Level

As a consequence of the above results, UVC appeared more effective to census families composed of small species, such as Pomacentridae, Pomacanthidae, Chaetodontidae and also small species of Labridae. Five out of the eight families composed of large species (Nemipteridae, Mullidae, Pomacanthidae, Labridae and Balistidae) were not detected in a significantly different way by the two techniques. However, a higher SR and greater density of large Serranidae species was observed with UVC. This could be explained by the fact that most individuals observed were smaller than 20 cm, and thus difficult to identify and count from videos.

Morevover, the study area has previously been subject to high fishing pressure, and large individuals are still quite scarce, compared to other MPAs in New Caledonia, and the densities observed for Serranidae were very low with both techniques (mean STAVIRO = 0.01 ind/10 m^2^ and mean UVC = 0.03 ind/10 m^2^).

In contrast, significantly greater densities of Scaridae and Acanthuridae were recorded with STAVIRO than with UVC (no significant difference in SR for these families). The greater densities of Acanthuiridae and Scaridae are mostly due to a higher abundance of *Zebrasoma* spp. and *Ctenochaetus* spp. not targeted in New Caledonia, and of juveniles of Scaridae.

### Differences in Detecting Community Structures

Three species assemblages were identified from UVC data: an intermediate reef assemblage characterized by lagoonal species, and two inner barrier reef assemblages characterized by species associated with live coral for the first one, and with rock, rubble and algal cover for the second one. These results were in accordance with two other UVC-based studies in the same region. First, Sarramégna [74] showed that substrate characteristics were the primary factor explaining fish assemblage structure, followed by the inshore-offshore gradient and finally by protection status. Second, Wantiez et al. [75] found that assemblages were organized along a nearshore-offshore gradient, but with a first explanatory factor being the protection status. In contrast, fish assemblages could not be discriminated according to reef type with the STAVIRO data. This is because in the 26 stations sampled in this study, the species that characterize the assemblage of the intermediate reef from UVC data were all small species, which as shown previously were not as well detected with the STAVIRO protocol used in the present comparative study. However, as explained above, the STAVIRO technique was not implemented with the usual protocol involving a high level of spatial replication. Studies using this regular STAVIRO protocol for monitoring fish assemblages are currently being conducted by some of the authors.

### Implications of Techniques

Even though both techniques used the same protocol, they may have different influences on the observations. Firstly, distances were not evaluated in a similar way. For STAVIRO, distances on footage were estimated using a database of screenshots of plastic fish silhouettes of different sizes, colours and distances from the camera. They were thus not measured in the field. On the other hand, for UVC a measuring tape set on the sea bottom before performing the counts was used to validate size and distance estimates. Therefore, the distances estimated from image analysis were less precise than those estimated by UVC. With regard to small species, differences between observations were more likely to be due to the observation radius used by each technique (3 m for UVC and 5 m for STAVIRO). For large species, no significant differences in densities were observed within the maximum range of observation (maximum visibility limited to 10 m) suggesting that differences in the surface area sampled were not significant.

Secondly, even if a particular attention was given to the direction of fish movement with respect to camera orientation, there is a risk of double counting inherent to video techniques, which is greatly reduced with UVC. In the present study, the total number of individuals observed (all levels of species identification confounded) over the 26 stations only slightly differed between techniques, since 2124 individuals were observed from STAVIRO versus 1941 ind. from UVC, i.e. a relative difference of 9% (Table 1). Considering in addition that small species were counted on a larger radius from STAVIRO than from UVC, we would expect a much larger abundance observed from STAVIRO under the assumption of repeated double counting. This was obviously not the case according to our results.

Thirdly, for the video technique, additional 35 hours were necessary to analyze the 26 stations for all species and individuals present on the images within a maximum radius of 10 m. This additional time required for image analysis should be taken into account when designing a study of an area as it is different according to the diversity and habitat complexity, and depends on the person analyzing footage. This time can also be reduced by taking into account a predefined list of species depending on the aim of the study. The STAVIRO protocol for spatial survey is generally based on a subset of species (26 families) and cost-efficiency issues of the technique were discussed in Pelletier et al. [30].

Finally, we did not carry out any comparison of fish size estimates between UVC and STAVIRO since exact sizes cannot be estimated with the STAVIRO technique (single camera). Size estimation was not central in the development of the STAVIRO system which gave priority to light portable systems aimed at highly replicated designs. For the purpose of monitoring major changes in fish assemblages, abundances per size class (small, medium and large) were thus preferred. Estimating individual size can be achieved from stereo-video systems [21] or from UVC to provide more precise biomass estimates based on known length-weight relationships, e.g. [34].

## Conclusion

Our study shows that from the 26 paired stations sampled on the reef slope; (i) UVC and STAVIRO did not detect the same fish assemblages; (ii) they did not significantly differ for large species (in both species richness and density); (iii) UVC detected more small species (for both species richness and density); (iv) STAVIRO detected a higher density of target species; and (v) only UVC detected differences in fish assemblages according to reef type. Main results of the present study were summarized in table 4.

**Table 4:** Synthesis of results obtained in the present study: comparison of observations performed on 26 paired stations on reef slopes by UVC and STAVIRO.

Metrics	Results
Overall fish population	SR and density : UVC>STAVIRO
Small species	SR and density : UVC>STAVIRO
Large species	SR and density : UVC = STAVIRO
Non-target species	SR and density: UVC>STAVIRO
Target species	SR : UVC = STAVIRO; density : STAVIRO>UVC
***Main families observed in the area studied***	
Small Serranidae	SR and density : UVC = STAVIRO
Nemipteridae	SR and density : UVC = STAVIRO
Mullidae	SR and density : UVC = STAVIRO
Large Pomacanthidae	SR and density : UVC = STAVIRO
Large labridae	SR and density : UVC = STAVIRO
Balistidae	SR and density : UVC = STAVIRO
Large Serranidae	SR and density : UVC>STAVIRO
Pomacentridae	SR and density : UVC>STAVIRO
Small Labridae	SR and density : UVC>STAVIRO
Scaridae	SR : UVC = STAVIRO; density : STAVIRO>UVC
Acanthuridae	SR : UVC = STAVIRO; density : STAVIRO>UVC
Chaetodontidae	SR : UVC = STAVIRO; density : UVC>STAVIRO
Small Pomacanthidae	SR : UVC = STAVIRO; density : UVC>STAVIRO

In the table. “>” and “<” correspond to significant difference between techniques with p<0.05 and “ = ” correspond to results of no significant difference (p>0.05) (see text for details on tests).

In the present study, the location of the observations was dictated by the UVC sampling design. During field work, a large number of unpaired stations were also deployed in other habitats: i) fringing reefs and sea grass beds (UVC and STAVIRO); ii) reef flats (UVC); and other soft bottoms (STAVIRO). Indeed, the two techniques may be coupled to survey a much larger area within a short period of time. UVC may focus on reef fishes within complex habitats, and particularly where live coral cover is high (e.g. reef slope), or in very shallow areas such as reef flats, or when visibility is low for STAVIRO but sufficient for UVC. Over the same period of time, STAVIRO would enable carrying out a large number of stations focused on large and diver-averse species. In particular, back reef areas, soft bottoms, sea grass beds, and more generally the areas not covered by UVC due to time and depth constraints could then be surveyed. This survey methodology would considerably increase the spatial coverage and replication level of fish monitoring activities when biomass and size estimation is not central to assessment.

## Supporting Information

Table S1 Lists of species observed.
(PDF)

Table S2 Target species in New Caledonia.
(PDF)

Table S3 Species observed characterizing structure’s groups.
(PDF)
