# Supplementary material for: Complementarity of Rotating Video and Underwater Visual Census for Assessing Species Richness, Frequency and Density of Reef Fish on Coral Reef Slopes
Source: PLoS One. 2014 Jan 2;9(1):e84344. doi: 10.1371/journal.pone.0084344 (PMC3879308; doi:10.1371/journal.pone.0084344)
Supplement: Table S1 — Lists of species observed. (PDF) [file pone.0084344.s001.pdf]

Table S1. Lists of species observed.

A species was considered as “small” when the maximum species size was less than 30 cm. The mobility of each species was characterized as: “HM”: highly mobile species, “MO”: mobile species and “SE”: sedentary species; following Kulbicki et al. (unpublished data).

List of species observed with both techniques (88 species)

family	genus	species	size	mobility
Acanthuridae	*Acanthurus*	*blochii*	large	MO
Acanthuridae	*Acanthurus*	*nigrofuscus*	large	SE
Acanthuridae	*Acanthurus*	*olivaceus*	large	MO
Acanthuridae	*Acanthurus*	*xanthopterus*	large	HM
Acanthuridae	*Ctenochaetus*	*striatus*	large	SE
Acanthuridae	*Naso*	*unicornis*	large	MO
Acanthuridae	*Zebrasoma*	*scopas*	large	SE
Acanthuridae	*Zebrasoma*	*veliferum*	large	SE
Balistidae	*Pseudobalistes*	*fuscus*	large	SE
Balistidae	*Rhinecanthus*	*aculeatus*	large	SE
Balistidae	*Sufflamen*	*chrysopterum*	large	SE
Blenniidae	*Meiacanthus*	*atrodorsalis*	small	SE
Caesionidae	*Pterocaesio*	*tile*	large	HM
Chaetodontidae	*Chaetodon*	*auriga*	small	SE
Chaetodontidae	*Chaetodon*	*bennetti*	small	SE
Chaetodontidae	*Chaetodon*	*citrinellus*	small	SE
Chaetodontidae	*Chaetodon*	*ephippium*	small	SE
Chaetodontidae	*Chaetodon*	*flavirostris*	small	SE
Chaetodontidae	*Chaetodon*	*lunulatus*	small	SE
Chaetodontidae	*Chaetodon*	*mertensii*	small	SE
Chaetodontidae	*Chaetodon*	*pelewensis*	small	SE
Chaetodontidae	*Chaetodon*	*plebeius*	small	SE
Chaetodontidae	*Chaetodon*	*trifascialis*	small	SE
Chaetodontidae	*Chaetodon*	*ulietensis*	small	SE
Chaetodontidae	*Chaetodon*	*vagabundus*	small	SE
Chaetodontidae	*Heniochus*	*acuminatus*	small	SE
Gobiidae	*Amblygobius*	*phalaena*	small	SE
Labridae	*Anampses*	*neoguinaicus*	small	MO
Labridae	*Cheilinus*	*chlorourus*	large	SE
Labridae	*Cheilinus*	*trilobatus*	large	SE
Labridae	*Cheilinus*	*undulatus*	large	SE
Labridae	*Coris*	*aygula*	large	MO
Labridae	*Coris*	*dorsomacula*	large	SE
Labridae	*Coris*	*gaimard*	large	SE
Labridae	*Gomphosus*	*varius*	small	SE
Labridae	*Halichoeres*	*hortulanus*	small	SE
Labridae	*Halichoeres*	*trimaculatus*	small	MO
Labridae	*Hemigymnus*	*melapterus*	large	MO
Labridae	*Labroides*	*dimidiatus*	small	SE
Labridae	*Novaculichthys*	*taeniourus*	large	MO
Labridae	*Thalassoma*	*hardwicke*	small	MO

Labridae	*Thalassoma*	*lunare*	small	MO
Labridae	*Thalassoma*	*lutescens*	small	MO
Lethrinidae	*Monotaxis*	*grandoculis*	large	MO
Lutjanidae	*Lutjanus*	*fulviflamma*	large	MO
Lutjanidae	*Lutjanus*	*quinquelineatus*	large	MO
Monacanthidae	*Oxymonacanthus*	*longirostris*	small	SE
Mullidae	*Parupeneus*	*barberinoides*	large	HM
Mullidae	*Parupeneus*	*barberinus*	large	HM
Mullidae	*Parupeneus*	*multifasciatus*	large	MO
Mullidae	*Parupeneus*	*pleurostigma*	large	MO
Nemipteridae	*Scolopsis*	*bilineata*	large	MO
Nemipteridae	*Scolopsis*	*trilineata*	large	MO
Ostraciidae	*Ostracion*	*cubicus*	large	MO
Pinguipedidae	*Parapercis*	*clathrata*	small	SE
Pinguipedidae	*Parapercis*	*hexophtalma*	small	SE
Pomacanthidae	*Centropyge*	*bicolor*	small	SE
Pomacanthidae	*Centropyge*	*tibicen*	small	SE
Pomacentridae	*Abudefduf*	*sexfasciatus*	small	SE
Pomacentridae	*Amblyglyphidodon*	*curacao*	small	SE
Pomacentridae	*Amphiprion*	*akindynos*	small	SE
Pomacentridae	*Chrysiptera*	*biocellata*	small	SE
Pomacentridae	*Chrysiptera*	*rollandi*	small	SE
Pomacentridae	*Chrysiptera*	*taupou*	small	SE
Pomacentridae	*Dascyllus*	*aruanus*	small	SE
Pomacentridae	*Dascyllus*	*reticulatus*	small	SE
Pomacentridae	*Dascyllus*	*trimaculatus*	small	SE
Pomacentridae	*Pomacentrus*	*chrysurus*	small	SE
Pomacentridae	*Pomacentrus*	*coelestis*	small	SE
Pomacentridae	*Pomacentrus*	*moluccensis*	small	SE
Pomacentridae	*Stegastes*	*nigricans*	small	SE
Priacanthidae	*Priacanthus*	*hamrur*	large	MO
Scaridae	*Chlorurus*	*microrhinos*	large	HM
Scaridae	*Chlorurus*	*sordidus*	large	MO
Scaridae	*Hipposcarus*	*longiceps*	large	HM
Scaridae	*Scarus*	*altipinnis*	large	HM
Scaridae	*Scarus*	*chameleon*	large	MO
Scaridae	*Scarus*	*frenatus*	large	SE
Scaridae	*Scarus*	*ghobban*	large	HM
Scaridae	*Scarus*	*rivulatus*	large	MO
Scaridae	*Scarus*	*schlegeli*	large	MO
Serranidae	*Epinephelus*	*merra*	large	SE
Serranidae	*Plectropomus*	*laevis*	large	MO
Serranidae	*Pseudanthias*	*hypselosoma*	small	SE
Siganidae	*Siganus*	*argenteus*	large	HM
Chaetodontidae	*Forcipiger*	*flavissimus*	small	SE
Labridae	*Thalassoma*	*nigrofasciatum*	small	MO
Tetraodontidae	*Canthigaster*	*valentini*	small	SE

List of species only observed with UVC

76 species: 30 large + 46 small and 46 SE + 20 MO + 10 HM

family	genus	species	size	mobility
Acanthuridae	*Acanthurus*	*albipectoralis*	large	HM
Acanthuridae	*Acanthurus*	*dussumieri*	large	MO
Acanthuridae	*Acanthurus*	*pyroferus*	large	SE
Acanthuridae	*Acanthurus*	*thompsoni*	large	MO
Acanthuridae	*Naso*	*annulatus*	large	HM
Apogonidae	*Apogon*	*aureus*	small	SE
Apogonidae	*Apogon*	*cyanosoma*	small	SE
Apogonidae	*Cheilodipterus*	*macrodon*	small	SE
Apogonidae	*Cheilodipterus*	*quinquelineatus*	small	SE
Blenniidae	*Atrosalarias*	*holomelas*	small	SE
Blenniidae	*Ecsenius*	*bicolor*	small	SE
Blenniidae	*Plagiotremus*	*rhinorhynchos*	small	SE
Blenniidae	*Plagiotremus*	*tapeinosoma*	small	SE
Caesionidae	*Pterocaesio*	*trilineata*	large	HM
Carangidae	*Carangoides*	*orthogrammus*	large	HM
Chaetodontidae	*Chaetodon*	*melannotus*	small	SE
Chaetodontidae	*Chaetodon*	*speculum*	small	SE
Chaetodontidae	*Chaetodon*	*unimaculatus*	small	SE
Chaetodontidae	*Heniochus*	*chrysostomus*	small	SE
Chaetodontidae	*Heniochus*	*monoceros*	small	SE
Cirrhitidae	*Cirrhitichthys*	*falco*	small	SE
Cirrhitidae	*Paracirrhites*	*forsteri*	small	SE
Gobiidae	*Istigobius*	*rigilius*	small	SE
Haemulidae	*Diagramma*	*pictum*	large	MO
Haemulidae	*Plectorhinchus*	*lineatus*	large	MO
Labridae	*Anampses*	*caeruleopunctatus*	small	MO
Labridae	*Cheilio*	*inermis*	large	MO
Labridae	*Choerodon*	*anchorago*	large	MO
Labridae	*Choerodon*	*graphicus*	large	MO
Labridae	*Cirrhilabrus*	*punctatus*	small	SE
Labridae	*Halichoeres*	*margaritaceus*	small	MO
Labridae	*Halichoeres*	*marginatus*	small	SE
Labridae	*Macropharyngodon*	*meleagris*	small	SE
Labridae	*Pseudocheilinus*	*evanidus*	small	SE
Labridae	*Stethojulis*	*bandanensis*	small	MO
Labridae	*Stethojulis*	*notialis*	small	MO
Labridae	*Stethojulis*	*strigiventer*	small	MO
Labridae	*Thalassoma*	*amblycephalum*	small	SE
Lethrinidae	*Gnathodentex*	*aureolineatus*	large	SE
Lethrinidae	*Lethrinus*	*xanthochilus*	large	HM
Lutjanidae	*Lutjanus*	*fulvus*	large	MO
Lutjanidae	*Lutjanus*	*kasmira*	large	MO
Mullidae	*Parupeneus*	*ciliatus*	large	HM
Mullidae	*Parupeneus*	*cyclostomus*	large	HM
Nemipteridae	*Scolopsis*	*temporalis*	large	HM

Pinguipedidae	*Parapercis*	*australis*	small	SE
Pinguipedidae	*Parapercis*	*millepunctata*	small	SE
Pomacanthidae	*Centropyge*	*bispinosa*	small	SE
Pomacanthidae	*Pomacanthus*	*imperator*	large	SE
Pomacentridae	*Amblyglyphidodon*	*leucogaster*	small	SE
Pomacentridae	*Amphiprion*	*melanopus*	small	SE
Pomacentridae	*Chromis*	*lepidolepis*	small	SE
Pomacentridae	*Chromis*	*viridis*	small	SE
Pomacentridae	*Chrysiptera*	*rex*	small	SE
Pomacentridae	*Neopomacentrus*	*azysron*	small	SE
Pomacentridae	*Plectroglyphidodon*	*dickii*	small	SE
Pomacentridae	*Plectroglyphidodon*	*lacrymatus*	small	SE
Pomacentridae	*Pomacentrus*	*adelus*	small	SE
Pomacentridae	*Pomacentrus*	*amboinensis*	small	SE
Pomacentridae	*Pomacentrus*	*bankanensis*	small	SE
Pomacentridae	*Pomacentrus*	*lepidogenys*	small	SE
Pomacentridae	*Pomacentrus*	*pavo*	small	SE
Pomacentridae	*Pomacentrus*	*philippinus*	small	SE
Pomacentridae	*Pomacentrus*	*vaiuli*	small	SE
Pomacentridae	*Stegastes*	*punctatus*	small	SE
Scaridae	*Scarus*	*globiceps*	large	SE
Scaridae	*Scarus*	*niger*	large	MO
Scaridae	*Scarus*	*rubroviolaceus*	large	HM
Serranidae	*Epinephelus*	*maculatus*	large	SE
Serranidae	*Plectropomus*	*leopardus*	large	MO
Siganidae	*Siganus*	*corallinus*	large	SE
Siganidae	*Siganus*	*puellus*	large	MO
Siganidae	*Siganus*	*spinus*	large	MO
Siganidae	*Siganus*	*vulpinus*	large	MO
Sphyraenidae	*Sphyraena*	*jello*	large	HM
Synodontidae	*Synodus*	*dermatogenys*	small	MO

List of species only observed with STAVIRO

28 species: 24 large + 4 small and 6 SE + 16 MO + 6 HM

**family**	**genus**	**species**	**size**	**mobility**
Acanthuridae	*Naso*	*tonganus*	large	HM
Aulostomidae	*Aulostomus*	*chinensis*	large	MO
Caesionidae	*Caesio*	*caerulaurea*	large	HM
Carangidae	*Caranx*	*melampygus*	large	HM
Carcharhinidae	*Triaenodon*	*obesus*	large	MO
Chaetodontidae	*Chaetodon*	*lineolatus*	small	SE
Diodontidae	*Diodon*	*holocanthus*	large	MO
Diodontidae	*Diodon*	*hystrix*	large	MO
Fistulariidae	*Fistularia*	*commersonii*	large	HM
Kyphosidae	*Kyphosus*	*gp*	large	MO
Labridae	*Bodianus*	*bilunulatus*	large	MO
Labridae	*Coris*	*batuensis*	large	SE
Labridae	*Hologymnosus*	*annulatus*	large	MO
Lethrinidae	*Lethrinus*	*atkinsoni*	large	MO
Lethrinidae	*Lethrinus*	*Harak*	large	MO
Lethrinidae	*Lethrinus*	*lentjan*	large	MO
Lethrinidae	*Lethrinus*	*rubrioperculatus*	large	MO
Lutjanidae	*Macolor*	*niger*	large	MO
Monacanthidae	*Paraluteres*	*prionurus*	small	SE
Mullidae	*Parupeneus*	*spilurus*	large	HM
Pomacentridae	*Chromis*	*retrofasciata*	small	SE
Pomacentridae	*Neoglyphidodon*	*melas*	small	SE
Scaridae	*Scarus*	*flavipectoralis*	large	MO
Scaridae	*Scarus*	*oviceps*	large	SE
Scaridae	*Scarus*	*psittacus*	large	MO
Siganidae	*Siganus*	*doliatus*	large	MO
Siganidae	*Siganus*	*punctatus*	large	MO
Stegostomatidae	*Stegostoma*	*fasciatum*	large	HM
