# Supplementary material for: Complementarity of Rotating Video and Underwater Visual Census for Assessing Species Richness, Frequency and Density of Reef Fish on Coral Reef Slopes
Source: PLoS One. 2014 Jan 2;9(1):e84344. doi: 10.1371/journal.pone.0084344 (PMC3879308; doi:10.1371/journal.pone.0084344)
Supplement: Table S2 — Target species in New Caledonia. (PDF) [file pone.0084344.s002.pdf]

Table S2. Target species in New Caledonia

Family	Genus	species
Acanthuridae	*Acanthurus*	*achilles*
Acanthuridae	*Acanthurus*	*blochii*
Acanthuridae	*Acanthurus*	*dussumieri*
Acanthuridae	*Acanthurus*	*lineatus*
Acanthuridae	*Acanthurus*	*mata*
Acanthuridae	*Acanthurus*	*nigricans*
Acanthuridae	*Acanthurus*	*nigricauda*
Acanthuridae	*Acanthurus*	*olivaceus*
Acanthuridae	*Acanthurus*	*pyroferus*
Acanthuridae	*Acanthurus*	*sp.*
Acanthuridae	*Acanthurus*	*xanthopterus*
Acanthuridae	*Naso*	*annulatus*
Acanthuridae	*Naso*	*brachycentron*
Acanthuridae	*Naso*	*brevirostris*
Acanthuridae	*Naso*	*hexacanthus*
Acanthuridae	*Naso*	*lituratus*
Acanthuridae	*Naso*	*sp.*
Acanthuridae	*Naso*	*tuberosus*
Acanthuridae	*Naso*	*unicornis*
Acanthuridae	*Naso*	*vlamingii*
Carangidae	*Atule*	*mate*
Carangidae	*Carangoides*	*chrysophrys*
Carangidae	*Carangoides*	*dinema*
Carangidae	*Carangoides*	*ferdau*
Carangidae	*Carangoides*	*fulvoguttatus*
Carangidae	*Carangoides*	*orthogrammus*
Carangidae	*Caranx*	*lugubris*
Carangidae	*Caranx*	*melampygus*
Carangidae	*Caranx*	*papuensis*
Carangidae	*Caranx*	*sexfasciatus*
Carangidae	*Decapterus*	*russelli*
Carangidae	*Elagatis*	*bipinnulata*
Carangidae	*Gnathanodon*	*speciosus*
Carangidae	*Pseudocaranx*	*dentex*
Carangidae	*Selar*	*crumenophthalmus*
Chanidae	*Chanos*	*chanos*
Clupeidae	*ge.*	*sp.*
Clupeidae	*Herklotsichthys*	*quadrimaculatus*
Clupeidae	*Spratelloides*	*sp.*
Gerreidae	*Gerres*	*oyena*
Haemulidae	*Diagramma*	*pictum*
kyphosidae	*Kyphosus*	*cinerascens*
kyphosidae	*Kyphosus*	*sp.*
kyphosidae	*Kyphosus*	*vaigiensis*
Labridae	*Bodianus*	*perditio*
Labridae	*Cheilinus*	*undulatus*
Lethrinidae	*Gymnocranius*	*euanus*
Lethrinidae	*Gymnocranius*	*grandoculis*
Lethrinidae	*Gymnocranius*	*sp.*
Lethrinidae	*Lethrinus*	*atkinsoni*
Lethrinidae	*Lethrinus*	*genivittatus*
Lethrinidae	*Lethrinus*	*harak*
Lethrinidae	*Lethrinus*	*lentjan*
Lethrinidae	*Lethrinus*	*miniatus*
Lethrinidae	*Lethrinus*	*nebulosus*
Lethrinidae	*Lethrinus*	*obsoletus*
Lethrinidae	*Lethrinus*	*rubrioperculatus*
Lethrinidae	*Lethrinus*	*variegatus*
Lutjanidae	*Aphareus*	*furca*
Lutjanidae	*Aprion*	*virescens*
Lutjanidae	*Lutjanus*	*adetii*
Lutjanidae	*Lutjanus*	*argentimaculatus*
Lutjanidae	*Lutjanus*	*sebae*
Lutjanidae	*Lutjanus*	*vitta*
Mugilidae	*ge.*	*sp.*
Mugilidae	*Valamugil*	*sp.*
Mullidae	*Parupeneus*	*barberinus*
Priacanthidae	*Priacanthus*	*hamrur*
Scaridae	*Bolbometopon*	*muricatum*
Scaridae	*Calotomus*	*carolinus*
Scaridae	*Cetoscarus*	*bicolor*
Scaridae	*Chlorurus*	*microrhinos*
Scaridae	*Chlorurus*	*sordidus*
Scaridae	*ge.*	*sp.*
Scaridae	*Hipposcarus*	*longiceps*
Scaridae	*Leptoscarus*	*vaigiensis*
Scaridae	*Scarus*	*altipinnis*
Scaridae	*Scarus*	*chameleon*
Scaridae	*Scarus*	*dimidiatus*
Scaridae	*Scarus*	*flavipectoralis*
Scaridae	*Scarus*	*forsteni*
Scaridae	*Scarus*	*frenatus*
Scaridae	*Scarus*	*ghobban*
Scaridae	*Scarus*	*globiceps*
Scaridae	*Scarus*	*longipinnis*
Scaridae	*Scarus*	*niger*
Scaridae	*Scarus*	*oviceps*
Scaridae	*Scarus*	*psittacus*
Scaridae	*Scarus*	*rivulatus*
Scaridae	*Scarus*	*rubroviolaceus*
Scaridae	*Scarus*	*schlegeli*
Scaridae	*Scarus*	*sp.*
Scaridae	*Scarus*	*spinus*

Scombridae	*Euthynnus*	*affinis*
Scombridae	*Grammatorcynus*	*bilineatus*
Scombridae	*Rastrelliger*	*kanagurta*
Scombridae	*Scomberomorus*	*commerson*
Serranidae	*Anyperodon*	*leucogrammicus*
Serranidae	*Cephalopholis*	*argus*
Serranidae	*Cephalopholis*	*leopardus*
Serranidae	*Cephalopholis*	*miniata*
Serranidae	*Cephalopholis*	*sonnerati*
Serranidae	*Cephalopholis*	*urodeta*
Serranidae	*Cromileptes*	*altivelis*
Serranidae	*Epinephelus*	*areolatus*
Serranidae	*Epinephelus*	*coioides*
Serranidae	*Epinephelus*	*cyanopodus*
Serranidae	*Epinephelus*	*fuscoguttatus*
Serranidae	*Epinephelus*	*hexagonatus*
Serranidae	*Epinephelus*	*howlandi*
Serranidae	*Epinephelus*	*lanceolatus*
Serranidae	*Epinephelus*	*macrospilos*
Serranidae	*Epinephelus*	*maculatus*

Serranidae	*Epinephelus*	*malabaricus*
Serranidae	*Epinephelus*	*merra*
Serranidae	*Epinephelus*	*ongus*
Serranidae	*Epinephelus*	*polyphekadion*
Serranidae	*Epinephelus*	*rivulatus*
Serranidae	*Epinephelus*	*tauvina*
Serranidae	*Plectropomus*	*leopardus*
Serranidae	*Variola*	*louti*
Siganidae	*Siganus*	*argenteus*
Siganidae	*Siganus*	*canaliculatus*
Siganidae	*Siganus*	*corallinus*
Siganidae	*Siganus*	*doliatus*
Siganidae	*Siganus*	*lineatus*
Siganidae	*Siganus*	*puellus*
Siganidae	*Siganus*	*punctatus*
Siganidae	*Siganus*	*spinus*
Siganidae	*Siganus*	*vermiculatus*
Siganidae	*Siganus*	*vulpinus*
Sillaginidae	*Sillago*	*sp.*
Sparidae	*Acanthopagrus*	*berda*
