# Supplementary material for: Complementarity of Rotating Video and Underwater Visual Census for Assessing Species Richness, Frequency and Density of Reef Fish on Coral Reef Slopes
Source: PLoS One. 2014 Jan 2;9(1):e84344. doi: 10.1371/journal.pone.0084344 (PMC3879308; doi:10.1371/journal.pone.0084344)
Supplement: Table S3 — Species observed characterizing structure’s groups. (PDF) [file pone.0084344.s003.pdf]

Table S3. Species observed characterizing structure's groups.

A species was considered as "small" when the maximum species size was less than 30 cm. The mobility of each species was characterized as: "HM": highly mobile species, "MO": mobile species and "SE": sedentary species; following Kulbicki et al. (unpublished data).

B1 = 1st cluster on the barrier reef; I = cluster on the intermediate reef; B2 = 2nd cluster on the barrier reef

family	genus	species	size	mobility	Groups from clustering analysis	Observed with technique
Acanthuridae	*Ctenochaetus*	*striatus*	large	SE	B1+B2	UVC and STAVIRO
Acanthuridae	*Zebrasoma*	*scopas*	large	SE	B1	
Caesionidae	*Pterocaesio*	*tile*	large	HM	B1	
Chaetodontidae	*Chaetodon*	*auriga*	small	SE	B1+B2	
Chaetodontidae	*Chaetodon*	*ulietensis*	small	SE	B1+B2	
Mullidae	*Parupeneus*	*multifasciatus*	large	MO	B1+B2	
Pomacentridae	*Amblyglyphidodon*	*curacao*	small	SE	B1	
Pomacentridae	*Chrysiptera*	*taupou*	small	SE	B1	
Pomacentridae	*Dascyllus*	*aruanus*	small	SE	B2	
Pomacentridae	*Pomacentrus*	*coelestis*	small	SE	B2	
Scaridae	*Chlorurus*	*microrhinos*	large	HM	B1	
Siganidae	*Siganus*	*argenteus*	large	HM	B1	
Acanthuridae	*Acanthurus*	*dussumieri*	large	MO	B1	UVC
Blenniidae	*Atrosalarias*	*holomelas*	small	SE	I	
Labridae	*Cirrhilabrus*	*punctatus*	small	SE	I	
Labridae	*Halichoeres*	*margaritaceus*	small	MO	I	
Labridae	*Thalassoma*	*amblycephalum*	small	SE	B1+B2	
Lethrinidae	*Lethrinus*	*xanthochilus*	large	HM	B1+B2	
Lutjanidae	*Lutjanus*	*kasmira*	large	MO	B1	
Mullidae	*Parupeneus*	*cyclostomus*	large	HM	B1+B2	
Pomacentridae	*Amblyglyphidodon*	*leucogaster*	small	SE	B1	
Pomacentridae	*Pomacentrus*	*pavo*	small	SE	I	
Pomacentridae	*Pomacentrus*	*philippinus*	small	SE	B1	
Serranidae	*Epinephelus*	*maculatus*	large	SE	B1+B2	
Sphyraenidae	*Sphyraena*	*jello*	large	HM	B1	
